# Effect of Injectate Viscosity on Epidural Distribution in Lumbar Transforaminal Epidural Steroid Injection

**DOI:** 10.1155/2019/2651504

**Published:** 2019-03-03

**Authors:** Jongwoo Choi, Nackhwan Kim, Matthew Smuck, Sang-Heon Lee

**Affiliations:** ^1^Graduate School, College of Medicine, Korea University, Seoul, Republic of Korea; ^2^Spine Center, Korea University Anam Hospital, Seoul, Republic of Korea; ^3^Department of Orthopaedic Surgery, Stanford University Medical Center, Stanford, CA, USA

## Abstract

**Introduction:**

There is no report on the effect of injectate viscosity on epidural distribution for lumbar transforaminal epidural steroid injections (L-TFESIs). The aim of this study was to evaluate the influence of injectate viscosity on the volume needed to reach specific landmarks in L-TFESIs.

**Methods:**

A prospective, randomized, comparative human study involving 118 patients undergoing L-TFESIs was conducted. The study subjects were divided into two groups by a random selection method: raw viscosity group (RV, *n*=58) and low viscosity group (LV, *n*=60). Contrast volumes were recorded as the contrast flow reached specific anatomical landmarks under fluoroscopic guidance.

**Results and Discussion:**

The possibility of delivering the injectate to each landmark showed a positive correlation with the amount and a negative correlation with the viscosity of the injectate. However, for landmarks at the medial aspect of the superior pedicle of the corresponding level of injection and for those beyond the spinous process over the contralateral spinal segment, the influence of viscosity was not statistically significant.

**Conclusion:**

The epidural distribution of the contrast agent through the transforaminal approach was most affected by the injectate volume and was also partly affected by the viscosity.

## 1. Introduction

The physiological pain associated with radicular pain, which is often caused by herniated nucleus pulposus (HNP), is due to ectopic discharge from the dorsal root or its ganglion [1]. The clinical features of radicular pain in the lumbosacral region are that it is well-presented, traveling along the length of the lower limb, and the pain area is relatively narrow compared to referred pain. According to Bogduk, when adhering to this definition, the prevalence rate is approximately 12% or less [2, 3].

For treating a lumbar HNP, which is the most common cause of radicular pain, a therapeutic injectate must be placed at the interface where the disc compromises the centrally transiting nerve. The procedure followed by conventional lumbar transforaminal epidural steroid injections (L-TFESIs), of injecting where the spinal nerve exits the level below the HNP, directs the flow along the spinal nerve, superiorly to the transiting nerve-disc interface. Sometimes, the conventional L-TFESIs are performed at the level above the central HNP, where the flow is directed inferiorly to the disc-nerve interface [4]. To reach the pathological site below the level of HNP, the injectate should spread to or beyond the superior disc, and to reach that above the level of HNP, it should spread to or below the inferior disc. Since the flow is believed to travel mostly ipsilaterally, the injection should be performed on the side of pain and pathology [5].

In clinical practice, the epidural distribution of the injectate is determined through the contribution pattern of a contrast agent injected just before a therapeutic injection. As steroid solutions, the components of therapeutic injectates have a lower viscosity than the contrast agent and are expected to spread to the same locations as the contrast, if not beyond them. Furman et al. showed that quantification of the injectate volume is needed to reach specific landmarks in the lumbar spine [4]. We expect that an injectate with a lower viscosity than the contrast would spread farther than the contrast agent. If less amount of medication can reach the same target site, a physician may maximally concentrate the steroid solution at the suspected pathological site to optimize the therapeutic efficacy of L-TFESIs.

The aim of this study is to evaluate the influence of injectate viscosity on the volume needed to reach specific landmarks in L-TFESIs.

## 2. Materials and Methods

### 2.1. Subjects

After the protocol of this study was approved from the Institutional Review Committee, patients over 20 years of age with low back and lower extremity pain who visited the outpatient clinic during a period of nine months were recruited and provided informed consent. The symptoms and signs of subjects with low back and lower extremity pain had been confirmed by precise physical examination, magnetic resonance images of the lumbosacral spine, and electrophysiologic studies, and finally diagnosed as lumbosacral radicular pain related to HNP by three physicians, two spine intervention specialists, and one radiology specialist. We recommended the L-TFESI to patients with little or no improvement of pain after least one month of conservative management, including physical therapy or oral analgesics. The following clinical history or findings were excluded: lumbosacral spine surgery, malignancy, inflammatory disease, severe deformity, overt upper motor neuron signs, multiple lower motor neuron signs, spinal cord injury, severe medication allergy, or pregnancy (suspected or confirmed). Subjects were divided into two groups by a random selection method: raw viscosity group (RV) and low viscosity group (LV). Patients were classified into the following three types: lumbosacral disc herniation, central spinal stenosis (foraminal stenosis excluded), and both.

### 2.2. Intervention and Injectate

The two intervention specialists (JWC and SHL) determined the appropriate injection level based on the patients' clinical histories and imaging studies. L-TFESIs were performed on the assigned patients with 5 mL of diluted or raw contrast agent using real-time fluoroscopy to ensure target flow and the absence of vascular or subdural flow. The procedures were all performed according to the International Spine Intervention Society guidelines [6]. A 23-gauge spinal needle was used, and the final needle tip position was the “safety triangle,” near 6o'clock direction of the pedicle on the ipsilateral oblique view and about 1 mm dorsal to the posterolateral vertebral body within the foraminal space on the lateral view fluoroscopically. A 5.0 mL syringe and extension tube system, which was utilized for injection, was filled and primed with diluted or raw contrast. The contrast agent was slowly injected under biplanar fluoroscopic guidance at a constant rate of 0.5–1.0 mL/sec, monitored manually. On the anteroposterior (AP) view, the contrast volumes were recorded as the contrast flow reached specific anatomical landmarks following:

1. The medial aspect of the superior pedicle of the corresponding level of injection (PED)
2. The superior aspect of the superior intervertebral disc of the corresponding level of injection (SIVD)
3. The inferior aspect of the inferior intervertebral disc of the corresponding level of injection (IIVD)
4. Both the SIVD and IIVD (BIVD)
5. Beyond the midline, spinous process, of the contralateral spinal segment (MID)

The contrast agent Omnipaque 300 (GE Healthcare, Shanghai, China) with an iodine concentration of 300 mg/mL was diluted with NaCl 0.9% to a dilution of 50%. The RV and LV groups were administered 5.0 mL of the raw contrast and 5.0 mL of the 50% diluted contrast, respectively. When the contrast reached the above anatomical landmarks, the total volume of contrast was recorded.

### 2.3. Analysis and Statistics

Before injection, the raw and diluted contrasts were prepared and filled in syringes by physicians who were not aware of the concentration (raw or 50% dilution). The two types of syringes bore the same color and the same amount. After the procedure, three physicians retrospectively reviewed the blinded images and determined again if the contrast had reached the landmarks. In order to analyze the final data, we selected the images, the results of which were agreed upon by all three physicians. Statistical analysis was performed to evaluate if there was a statistically significant difference in the demographic data of the two groups by the independent Student's *t*-test or Pearson's chi-squared test. The multiple linear regression model was used to analyze the effect of the amount and the viscosity to reach to the landmarks, using SPSS 12.0 KO software for Windows (SPSS Korea Datasolution Inc., Seoul, Korea). The five different outcome variables of the landmarks and two explanatory variables of viscosity and amount were presented in regression analysis. The significance was determined at *p* value <0.01.

## 3. Results

Two groups were similar regarding the basic patient characteristics (Table 1).

**Figure 1:**
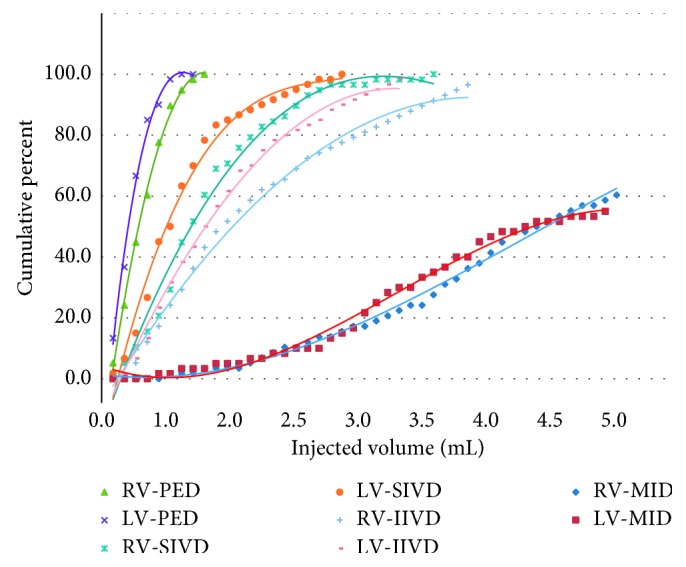
Dot plots and trendlines reaching specific landmarks in each group. RV: group of raw viscosity, LV: group of low viscosity, PED: the medial aspect of the superior pedicle of the corresponding level of injection, SIVD: the superior aspect of the superior intervertebral disc of the corresponding level of injection, IIVD: the inferior aspect of the inferior intervertebral disc of the corresponding level of injection, MID: beyond the midline, spinous process, of the contralateral spinal segment.

Table 2 shows the injected amount that reached the PED fluoroscopically, and the number and the cumulative percentage of eligible subjects were calculated according to the amount. If we interpret this “cumulative percentage” as another aspect, the possibility that each amount reaches the landmarks can be considered. The correlation between this cumulative percentage and the injected amount was displayed as a graph, and the two groups were compared. The same process was performed based on each landmark (Figure 1).

A multiple linear regression model was used to analyze the effect of the amount and the viscosity on reaching the landmarks. We have assumed the “cumulative percentage” to be the possibility of reaching the specific landmark in a specific amount and analyzed how the amount and the viscosity affect this possibility. The possibility to deliver the injectate to each landmark showed a positive correlation with the amount and negative correlation with the viscosity. However, in the case of PED and MID, the influence of viscosity was not statistically significant (Table 3).

**Figure 2:**
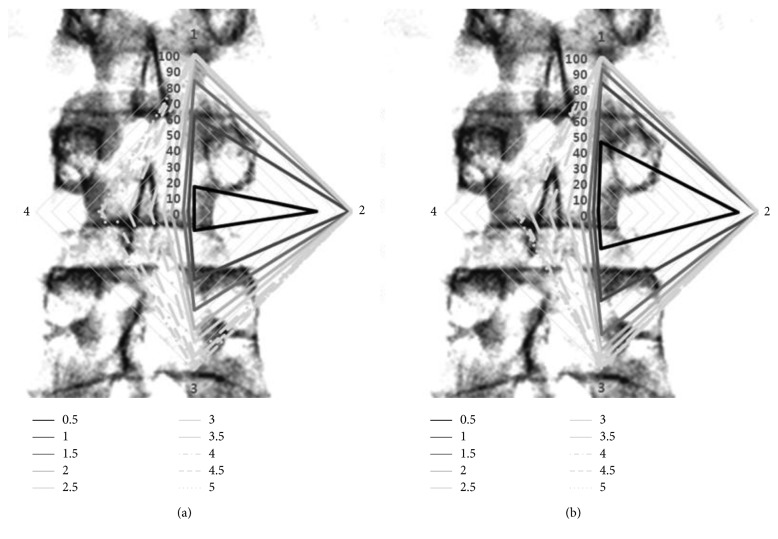
Radar plots over a sample of lumbar spine anteroposterior view, which represent the possibility of reaching by amount in the two groups. (a) is the result of the RV group, and (b) is the result of the LV group. “1,” upper vertex, is corresponding to the landmark of SIVD. “2,” right vertex, is to PED. “3,” lower vertex, is to IIVD, and “4,” left vertex, is to MID. For example, the rhombus of a solid black line indicates a point in which a 0.5 mL infusion can be reached. This rhombus is wider at B than at A. This means that the injectate with a lower viscosity at the same amount can be more distantly distributed. In the radar plot based on anatomic landmarks set in this study, each vertex represents a reachable point, and the area of rhombus does not reflect the actual wideness of distribution.

Based on the multiple regression analysis, the possibility of reaching by amount was calculated and the results of the two groups were expressed by a radar plot (Figure 2). The upper part of the rhombus shows SIVD, the right side shows PED, the lower part shows IIVD, and the left side shows MID, which is contrasted with the spine AP view.

## 4. Discussion

Therapeutic L-TFESIs are an integral part of comprehensive and conservative care for radicular pain [6–13]. The theoretical goal is to place a mixture of concentrated steroid and anesthetic solution at the pathological site or along the dorsal root ganglion [7, 14]. Therapeutic L-TFESIs result in the flow of medication from the needle tip to the dorsal root ganglion, medial to the pedicle, and into the epidural space [15]. Many efforts have been made to optimize the therapeutic effect of L-TFESIs by concentrating the medication flow to the pathological site relevant to the patient's clinical and radiographic presentation. The proposed mechanisms of pain relief include decreasing inflammatory mediators, diminishing edema, interrupting afferent impulses, and possibly providing membrane stabilization [4]. Using the smallest possible volume of medication by increasing its concentration would be expected to optimize the therapeutic effect at the pathological site.

The pathological site is thoroughly determined by clinical, radiographic, and electrodiagnostic studies, as part of a comprehensive evaluation because there are many suspected pain generators and structural complexities in the spinal segment. In case of radicular pain, the potential sites at which the L4 spinal nerve and root can be compromised are an L3/4 central stenosis or a central HNP, an L4/5 lateral recess or foraminal stenosis may contact the exiting L4 nerve root or ganglion, and a far lateral L4/5 HNP may affect the existing L3 spinal nerve. Furthermore, other common causes for radicular pain include compression from zygapophysial joints, synovial cysts, epidural lipomatosis, and/or postsurgical epidural scarring [16].

Measuring the reach of the landmark according to the amount of injectate is a very useful method for studying contrast agent and medication distribution and has the advantage of minimizing intervention in the existing treatment process for clinical research. Through the cumulative measurement of the injected volume reaching the target landmark, the likelihood that a particular volume of injectate will reach the specified anatomical location can be estimated. This interpretation is expected to have more statistical persuasiveness if the number of subjects in whom each amount reaches the target landmarks is normally distributed.

The summary of these results is as follows. The epidural distribution of the contrast agent through the transforaminal approach was most affected by the injectate volume and was also affected by the viscosity. Although the effect of viscosity was not statistically significant for reaching the nearest landmark, PED, the possibility of reaching the SIVD and IIVD was statistically significant. In addition, in the case of MID, for the landmark that required a relatively large amount of injectate, the effect of viscosity on the reachability was not statistically significant.

The epidural space is a potential space, which is formed by the amount of injection, and the injectate is distributed along the space. While the space is forming a volume, a certain amount of pressure will be generated, such that the injectate can be distributed farther than the position of injection. The amount is a factor that promotes the distribution of the injectate because it directly affects the volume, and the viscosity that contributes to maintaining the volume is a factor that hinders the distribution.

These findings are clinically relevant in two respects. A small distribution with a relatively high viscosity injectate is less likely to cover multiple lesions with a single injection, whereas when the lesion is singularly injected with a small amount, the injectate can stay in the lesion selectively. In other words, if a highly viscous injectate is applied in the minimum amount, the selectivity can be greatly increased.

When the injected amount was increased, the influence of viscosity was increased. There was some positive correlation between the amount of injectate and the influence of viscosity. This occurs due to physical reasons but may also be due to differences in the diffusion rate between two viscosity injectates in the epidural space. We did not perform a time-serial measurement because there was no purpose of confirming the final distribution of the injectate. However, it is assumed that the diffusion time would be longer for higher viscosity injectates. Missing data on the distribution over time is a limitation of this study, and further research is needed.

The type of disease was not closely controlled, which is the limitation of this study. The distribution of injectate in HNP and spinal stenosis may differ. However, the focus of this study was not to analyze the viscosity effects of injectate by disease type and therefore did not unify the type. The reason for excluding foraminal stenosis is that the infusion is expected to have a significant effect from entering the epidural space across the foramen. Additional research is needed to determine the effect of injectate viscosity with various pathoanatomical conditions.

In our study, the rate of transfer to the contralateral epidural space was similar for the two groups. This seems to be more influenced by the amount. One hypothesis is that the pressure in the central and the ventral epidural space is higher than that in the lateral space, such that a certain threshold of pressure must be allowed for the injectate to cross over the midline. In other words, the distance to midline after injection is shortened to minimize the influence of viscosity, and it is considered that further increase of the pressure by the amount is required to pass the critical threshold of pressure. Additional research is necessary for clinical interpretation.

## 5. Conclusions

Dilution may be necessary to allow the contrast agent distribution to reflect the injectate distribution as closely as possible or it should be recognized that the injectate can be delivered to a somewhat larger area than the contrast agent distribution. To increase the selectivity of the injection, there is a limit on the minimum injected amount, so adjusting the viscosity will be a factor to consider. In addition, when the injection amount is increased, high viscosity can be a factor in causing pain because it can maintain the volume of the injection space and increase the pressure. For diagnostic purposes, local and selective injections may be possible, given the amount and the viscosity of injectate.

## Figures and Tables

**Table 1:** Basic characteristics of the two viscosity groups.

Demographic	RV (*n*=58)	LV (*n*=60)	*p* value^*∗*^
Age (year, mean ± SD)	55.2 ± 13.7	54.9 ± 15.9	0.931
Weight (kg, mean ± SD)	58.2 ± 7.3	59.2 ± 4.8	0.381
Height (cm, mean ± SD)	162.6 ± 6.6	163.5 ± 3.4	0.352
Sex (number, male:female)	38 : 20	41 : 19	0.745
History of smoking (%)	25.9	33.3	0.374
Diagnosis (number, herniation : stenosis : both)	38 : 17 : 3	38 : 19 : 3	0.962
Level of injection (number, L4 : L5)	12 : 46	14 : 46	0.729

RV: group of raw viscosity; LV: group of low viscosity; SD: standard deviation. ^*∗*^A statistically significant difference in the basic characteristics of the two groups is analyzed by the independent Student's *t*-test or Pearson's chi-squared test.

**Table 2:** Observed injected volumes and calculated cumulative percentage reaching the medial aspect of the superior pedicle of the corresponding level of injection in two groups.

Volume (mL)	Subjects (*n*)	Percentage	Cumulative percentage
*RV*			
0.1	3	5.17	5.17
0.2	11	18.97	24.14
0.3	12	20.69	44.83
0.4	9	15.52	60.34
0.5	10	17.24	77.59
0.7	7	12.07	89.66
0.8	3	5.17	94.83
1	2	3.45	98.28
1.2	1	1.72	100.00
Never^*∗*^	0	0.00	100.00
Total	58	100.00	
*LV*			
0.1	8	13.33	13.33
0.2	14	23.33	36.67
0.3	18	30.00	66.67
0.4	11	18.33	85.00
0.6	3	5.00	90.00
0.7	5	8.33	98.33
0.8	1	1.67	100.00
Never^*∗*^	0	0.00	100.00
Total	60	100.00	

RV: group of raw viscosity; LV: group of low viscosity. ^*∗*^Cases that do not reach the landmark fluoroscopically despite any amount.

**Table 3:** Multiple regression analysis of injected amount and viscosity for possibility of reaching the specific landmarks (*n*=118).

Possibility of reaching at the landmarks	Unstandardized coefficient (*p* value)		Adjusted *R* square
	Viscosity^†^	Amount (mL)	
Reaching at PED	−16.5 (0.051)	93.6 (<0.001)	0.796
Reaching at SIVD	−18.7 (<0.001)	38.9 (<0.001)	0.813
Reaching at IIVD	−14.1 (<0.001)	31.1 (<0.001)	0.873
Reaching at BIVD	−13.5 (<0.001)	33.4 (<0.001)	0.937
Reaching at MID	−1.5 (0.105)	14.5 (<0.001)	0.955

PED: the medial aspect of the superior pedicle of the corresponding level of injection; SIVD: the superior aspect of the superior intervertebral disc of the corresponding level of injection; IIVD: the inferior aspect of the inferior intervertebral disc of the corresponding level of injection; MID: beyond the midline, spinous process, of the contralateral spinal segment. ^†^The viscosity variable is a binary value: raw (100%) and low (50% dilution) variables.

## Data Availability

All significant data supporting the analysis in this study are contained in the tables and figures published within the paper.
